# Prophylaxis of Antifungal Drugs against Systemic Fungemia induced by Oral Candidiasis in Mice

**DOI:** 10.3390/cimb45020085

**Published:** 2023-02-04

**Authors:** Kazunori Ninomiya, Hiroki Katagiri, Hajime Hara, Kayoko Fukui, Maiko Haga-Tsujimura, Ken Nakahara, Kenjirou Nakamura

**Affiliations:** 1 Department of Pharmacology, The Nippon Dental University School of Life Dentistry at Niigata, 1-8 Hamaura-cho, Chuo-ku, Niigata 951-8580, Japan; 2 Advanced Research Center, The Nippon Dental University School of Life Dentistry at Niigata, 1-8 Hamaura-cho, Chuo-ku, Niigata 951-8580, Japan; 3 Department of Histology, The Nippon Dental University School of Life Dentistry at Niigata, 1-8 Hamaura-cho, Chuo-ku, Niigata 951-8580, Japan

**Keywords:** fungemia, prophylaxis, antifungal drugs, oral, systemic candidiasis, *Candida albicans*

## Abstract

Oral mucositis is highly prevalent among the elderly, for whom oral care is often difficult. Oral mucositis, such as candidiasis, can induce systemic fungemia. Antifungal prophylaxis may be useful in such cases to prevent systemic fungemia; however, studies on this are limited. The objective of this study was to demonstrate the effectiveness of antifungal prophylaxis to prevent systemic *Candida* dissemination compared to oral care using a mice model. Oral candidiasis was induced using chemotherapy and inoculation with *C. albicans in* 8-week-old male mice. Group A was given oral care, Group B was orally administered an antifungal drug, Group C was intravenously administered an antifungal drug, and Group D was used as the negative control group. Macroscopic features of the tongue surface, colony forming units (CFU) on the tongue, and blood culture for *C. albicans* were evaluated. CFU was significantly higher in Group A than in Groups B and C. The oral care group, but not the groups administered antifungal agents, showed significantly higher positive numbers of animals with *C. albicans* in the blood as compared to the control group, indicating the effectiveness of antifungal prophylaxis over oral care. Antifungal prophylaxis may be an option for the prevention of systemic fungemia in individuals with difficulty in oral care.

## 1. Introduction

Systemic fungemia (fungemia) can be induced by oral mucositis via hematogenous dissemination [1]. Several antifungal drugs are commercially available for the treatment of fungemia. Antifungal drugs are commonly administered to patients with fungemia, although prophylactic administrations are less recommended. The prophylactic administrations of antifungal drugs are strongly recommended only for immune-compromised patients, including in acute leukemia and myelodysplastic syndrome [2]. For patients with neutropenia, antifungal prophylaxis is only recommended for those expected to have <100 neutrophils/µL for >7 days [3]. No recommendation for prophylactic administration for immunocompetent patients to prevent fungemia has been shown.

The prevalence of oral candidiasis, a major oral mucositis, is high among elderly people due to their immune-compromised condition [4]. Oral care is one of the most effective methods to prevent oral candidiasis [5,6]; however, effective oral care is often difficult for elderly people with cognitive impairment, leading to the worsening of oral hygiene and, frequently, oral candidiasis. Oral candidiasis may induce opportunistic infections, such as fungemia and fungal pneumonia [7,8]. The prophylactic administration of antifungal drugs may be effective for elderly people experiencing difficulty with oral care. It is, however, still unknown how the prophylactic administration of antifungal drugs may be effective in preventing oral candidiasis-related fungemia. To the best of our knowledge, no studies have shown which preventive method is more effective in the prophylactic administration of antifungal drugs and oral care. 

We previously developed a mouse model with systemic hematogenous dissemination induced by oral candidiasis [1]. Candida albicans invades the surface of the tongue and blood vessels, leading to dissemination throughout the body in this immunosuppressed mice model. Oral care can impede the dissemination of Candida. Therefore, the present study aimed to investigate the effectiveness of the prophylactic administration of antifungal drugs to prevent Candida dissemination compared to oral care. 

## 2. Materials and Methods

### 2.1. Animals

Eight-week-old male ICR mice (CLEA, Tokyo, Japan) were randomly divided into the following four groups: oral care (Group A), oral administration of an antifungal drug (Group B), intravenous administration of an antifungal drug (Group C), and the negative control group, without microbial/drug administration and mucositis induction (Group D) (*n* = 10, each group). The mice were housed at room temperature (24 °C) with free access to food and water. Tetracycline hydrochloride (0.83 g/L; Wako Pure Chemical Industries, Tokyo, Japan) was added to the water for infection control. This study was approved by the Animal Experimentation Ethics Committee of The Nippon Dental University School of Life Dentistry at Niigata (Approval No. 201). 

### 2.2. Experimental Schedule

**Figure 1:**
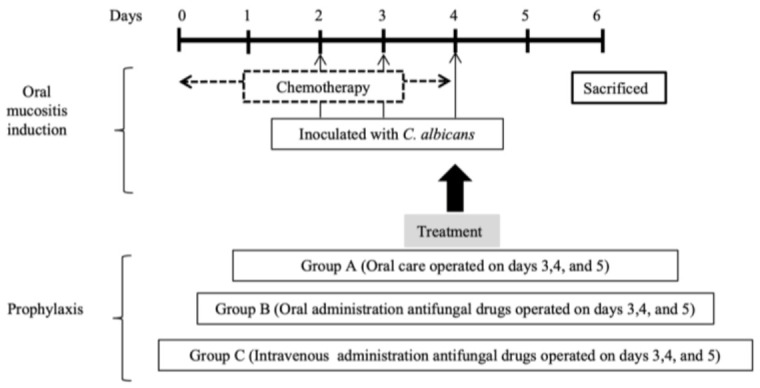
Experimental schedule.

In each group, chemotherapy and the induction of oral mucositis were carried out as previously described by Katagiri et al. [1], and inoculation with Candida albicans was performed as described by Takakura et al. [9]. Briefly, for chemotherapy, 7 mg/kg cisplatin on day 1 and 10 mg/kg 5-fluorouracil on days 1, 2, 3, and 4 were intraperitoneally injected into the mice. For C. albicans inoculation, 5.0 × 10^6^ cells/25 µL of the ATCC 48130 strain were orally administered at day 2, 3, and 5 of chemotherapy. Prophylactic treatment in Groups A, B, and C was performed on days 3, 4, and 5 (Figure 1). In Group A, the mouth was carefully cleaned with sterile cotton and saline until no removable stains remained in the entire mouth. In Group B, 2% miconazole gel was administrated orally at 60 mg/kg with sterile cotton (Mochida Pharmaceutical, Tokyo, Japan). In Group C, fluconazole 1 mg/mL (Pfizer, Tokyo, Japan) was administrated intravenously at 0.52 mg/kg. All mice were sacrificed on day 6 with a lethal dose of general anesthesia. At the same time, a blood sample for culture and polymerase chain reaction (PCR) was taken from the cardiac apex without any contamination. 

### 2.3. Colony Forming Units

At the time of sacrifice, tongues were collected with no contamination, weighed, and then washed with sterile saline. The tissue was then homogenized using a sterile micro-tube (BioMasher II, Tokyo, Japan). A volume of 100 μL of solution was then spread on Sabouraud dextrose agar plates (Nissui Pharmaceutical, Tokyo, Japan) and incubated at 30 °C for 48 h. The growth of C. albicans was confirmed with the naked eye and the colonies were counted.

### 2.4. Blood Culture

Collected blood was immediately injected into peptone yeast extract glucose liquid medium and incubated at 30 °C for 72 h. This medium was then spread on Sabouraud dextrose agar plates (Nissui Pharmaceutical) and incubated at 30 °C for 48 h. The plates were carefully observed for colonies and evaluated as positive or negative for C. albicans.

### 2.5. Multiplex PCR and Nested PCR

Positive colonies from blood culture were analyzed by multiplex PCR to determine if the presence of C. albicans was similar to the Candida that we inoculated in the mice. Nested PCR methods were conducted for blood and tissue (tongue, liver, and kidney) in Group D to confirm that C. albicans was not present in the mouse normal oral flora or in the environment or equipment during the experiment. DNA for nested PCR was purified using DNA purification kits (Norgen Biotek Thorold, ON, Canada). The primers were designed according to Bougnoux et al. for multiplex PCR and for nested PCR, as previously reported [9,10].

### 2.6. Statistical Analysis

The statistical significance of the colony forming units (CFU) in mouse tongues was evaluated using the Steel–Dwass multiple comparison test, using the statistics add-in for BellCurve for Excel (Social Survey Research Information, Tokyo, Japan). The blood culture results were analyzed using Fisher’s exact test in SPSS v25 (IBM, Armonk, NY, USA). A *p*-value < 0.05 was considered statistically significant.

## 3. Results

**Figure 2:**
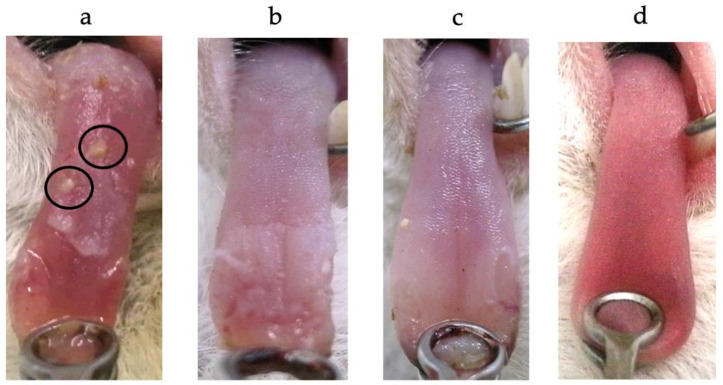
Macroscopic pictures of the tongue. (**a**) Group A: The presence of clinical oral candidiasis and food residue (circle). (**b**) Group B: A decrease in white patches on the tongue was observed compared to Group A. (**c**) Group C: The oral mucositis was decreased as compared to Group A and Group B. (**d**) Group D: The tongue of the negative control group.

**Figure 3:**
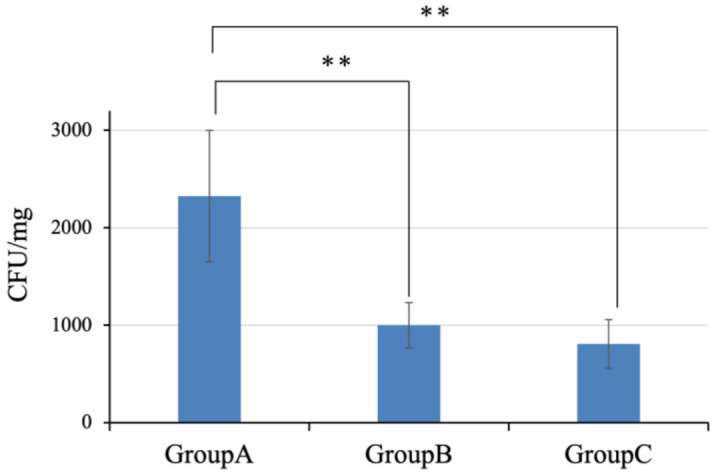
Colony forming Unit (CFU) counts in the tongue. The CFU counts were obtained in Group A. Between Groups A and B, Groups A and C were statistically significant (** *p* < 0.01). There was no significant difference between Groups B and C.

In this experiment, we first observed the effect of oral care on the reduction of Candida albicans on the surface of the tongue using an animal model. Macroscopically, no reduction of the white patch of Candida albicans was observed in the oral care compared to the non-oral care groups. Therefore, we examined how oral and intravenous administrations of antifungal drugs affected the reduction of the white patch compared to the oral care group. White patches on the tongue were confirmed in all the experimental groups, indicating oral candidiasis on the tongue (Figure 2). The amount of the white patches appeared to be increased in the order of Groups A > B > C. We confirmed more food residue in this group order. The tongue showed a mean (±SD) of 2325.03 ± 674.8 CFU/mg in group A, 998.72 ± 232.4 CFU/mg in Group B, and 807.16 ± 249.1 CFU/mg in Group C. CFU were significantly higher in group A than in Groups B and C (*p* < 0.01). A significant difference in CFU was not found between Groups B and C (Figure 3). 

**Figure 4:**
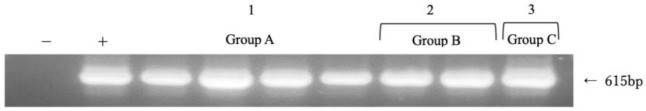
Positive results of multiplex PCR for blood cultures. The 615 bp band specifically identifies *Candida albicans* among *Candida* spp. Lane −: negative control (buffer), lane +: positive control (*C. albicans*).

**Figure 5:**
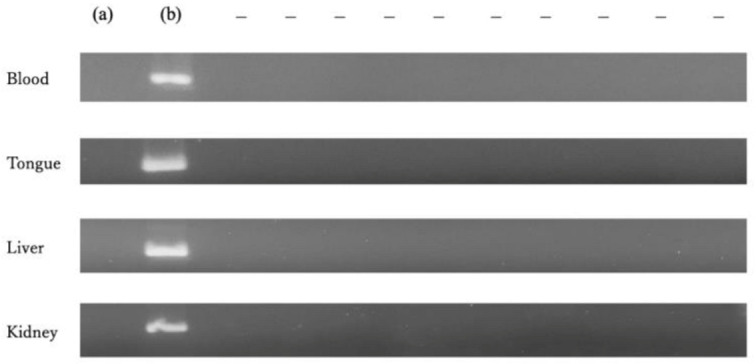
Results of nested PCR. Lane (**a**): negative control (buffer), lane (**b**): positive control (*C. albicans*); the lane to the right indicates positive (+) or negative (−). All specimens from the blood, the tongue, the liver, and the kidney were negative for *C. albicans*, indicating that there was no contamination of the experimental environment and experimental animals.

Blood culture showed positives for C. albicans in 4/10 mice in Group A, 2/10 in Group B, 1/10 in Group C, and 0/10 in the control group. There was a significant difference in the numbers of positives for C. albicans between Group A and the control group (*p* < 0.05), while no significant differences were found between Group B or C and the control group (Table 1). No significant difference was observed between Groups A and B or C. All specimens from blood cultures which we determined as positive were identified as the C. albicans strain by multiplex PCR (Figure 4). A nested PCR in Group D showed that all specimens from the blood, the tongue, the liver, and the kidney of the negative control group were negative for C. albicans (Figure 5).

## 4. Discussion

The present study demonstrated that the prophylactic administration of antifungal drugs was more effective at preventing oral candidiasis than oral care. Prophylactic administration may prevent systemic fungemia induced by oral candidiasis. Oral care is one of the most effective methods to prevent oral candidiasis [5]. Our animal experiment suggested that the prophylactic administration of antifungal drugs is an option for the prevention of fungemia in the elderly with immune-compromised conditions, especially with difficulty in oral care. Prophylactic administration, as well as the long-term administration of antifungal drugs, often leads to the development of resistant bacteria [11], and often generates harmful effects on kidney and liver function in the elderly [12,13,14,15]. Although prophylactic administration is more likely to be avoided in the elderly, it should be considered for the elderly with immune-compromised conditions, especially those having difficulty with oral care. 

We previously established a mouse model with systemic hematogenous dissemination induced by oral candidiasis [1]. We confirmed that *Candida albicans* invaded the surface of the tongue in the animal model. In this experiment, we first observed the effect of oral care on the reduction of *Candida albicans* on the surface of the tongue using this model. Macroscopically, no reduction of the white patch of *Candida albicans* was observed in the oral care compared to the non-oral care groups. Therefore, we examined how antifungal drugs affected the reduction of the white patch using different administration methods: oral and intravenous administration. Both oral and intravenous administration significantly reduced the white patches, indicating that antifungal drug administration may be more effective for the reduction of Candida than oral care.

We chose miconazole gel for oral administration and fluconazole for intravenous administration. Both miconazole and fluconazole are azole series antifungal agents, with the former and latter belonging to the imidazole and triazole groups, respectively [16,17]. Although both inhibit CYP3A4, a metabolizing enzyme, the inhibitory effect of the triazole group is weaker than that of the imidazole group [18]. Fluconazole has been used for systemic administration more than miconazole. There are many advantages for local application of miconazole for oral candidiasis [19]. These advantages include the fact that miconazole is more effective than nystatin for oral candidiasis, the relapse rate of miconazole oral gel may be lower than other types of antifungal agents, the broad-spectrum activity of miconazole, and most importantly, its lower development of chemo-resistance [19,20]. Furthermore, miconazole is effective against several species of *Candida* that are resistant to fluconazole [21]. Owing to the efficacy and safety of fluconazole, it is considered as the first-line treatment for treating localized and systemic *C. albicans* infections [22,23]. However, prolonged and repeated treatment with fluconazole can cause antifungal resistance. The prophylactic administration of fluconazole should be for short periods, and repeated prophylactic administrations should be avoided [24,25,26]. The adverse effects of using antifungal drugs include nephrotoxicity, hepatotoxicity, hypokalemia, bronchospasm, and acute drug reactions, among others. However, the careful monitoring of adverse effects and short-term prophylactic doses may minimize this effect. 

A blood culture showed that the positive number of animals with *C. albicans* was significantly more in Group A than in the negative control. No significant differences were found between Groups A and B or C, and between Group B or C and the control group. If a significant difference had occurred between Groups A and B or C, it could be strongly suggested that the effect of prophylactic administration of antifungal drugs against systemic fungemia is greater than the effect of oral care. Fewer animals with positive *C. albicans* in the blood of Groups B and C and the control group than Group A may suggest that prophylactic administration prevented systemic fungemia more than oral care did. Additional investigations are needed to confirm this.

In conclusion, the present study demonstrated that the prophylactic administration of antifungal drugs was more effective at preventing oral candidiasis than oral care. Prophylactic administration may prevent systemic fungemia induced by oral candidiasis. The prophylactic administration of antifungal drugs may be an option for the prevention of fungemia in the elderly with immune-compromised conditions, especially in those having difficulty with oral care. Further clinical investigations are needed to confirm this hypothesis.

## Figures and Tables

**Table 1:** Blood culture analysis.

	Group A	Group B	Group C	Negative Control
Positive animals/Total animals	4/10	2/10	1/10	0/10

## Data Availability

Available on request to the corresponding author.
